# Transient Transfection of a Wild-Type p53 Gene Triggers Resveratrol-Induced Apoptosis in Cancer Cells

**DOI:** 10.1371/journal.pone.0048746

**Published:** 2012-11-12

**Authors:** Danielly Cristiny Ferraz da Costa, Fabiana Alves Casanova, Julia Quarti, Maitê Santos Malheiros, Daniel Sanches, Patricia Souza dos Santos, Eliane Fialho, Jerson L. Silva

**Affiliations:** 1 Instituto de Bioquímica Médica, Universidade Federal do Rio de Janeiro, Rio de Janeiro, Brazil; 2 Instituto Nacional de Ciência e Tecnologia de Biologia Estrutural e Bioimagem, Universidade Federal do Rio de Janeiro, Rio de Janeiro, Brazil; 3 Instituto de Nutrição Josué de Castro, Universidade Federal do Rio de Janeiro, Rio de Janeiro, Brazil; Wake Forest University, School of Medicine, United States of America

## Abstract

Resveratrol is a promising chemopreventive agent that mediates many cellular targets involved in cancer signaling pathways. p53 has been suggested to play a role in the anticancer properties of resveratrol. We investigated resveratrol-induced cytotoxicity in H1299 cells, which are non-small lung cancer cells that have a partial deletion of the gene that encodes the p53 protein. The results for H1299 cells were compared with those for three cell lines that constitutively express wild-type p53: breast cancer MCF-7, adenocarcinomic alveolar basal epithelia A549 and non-small lung cancer H460. Cell viability assays revealed that resveratrol reduced the viability of all four of these cell lines in a dose- and time-dependent manner. MCF-7, A549 and H460 cells were more sensitive to resveratrol than were H1299 cells when exposed to the drug for 24 h at concentrations above 100 µM. Resveratrol also increased the p53 protein levels in MCF-7 cells without altering the p53 mRNA levels, suggesting a post-translational modulation of the protein. The resveratrol-induced cytotoxicity in these cells was partially mediated by p53 and involved the activation of caspases 9 and 7 and the cleavage of PARP. In H1299 cells, resveratrol-induced cytotoxicity was less pronounced and (in contrast to MCF-7 cells) cell death was not accompanied by caspase activation. These findings are consistent with the observation that MCF-7 cells were positively labeled by TUNEL following exposure to 100 µM resveratrol whereas H1299 cells under similar conditions were not labeled by TUNEL. The transient transfection of a wild-type p53-GFP gene caused H1299 cells to become more responsive to the pro-apoptotic properties of resveratrol, similarly to findings in the p53-positive MCF-7 cells. Our results suggest a possible therapeutic strategy based on the use of resveratrol for the treatment of tumors that are typically unresponsive to conventional therapies because of the loss of normal p53 function.

## Introduction

Cancer is a major public health concern worldwide, and the number of cancer-related deaths is expected to double in the next 50 years [1]. Epidemiological evidence suggests that dietary habits are important risk factors associated with cancer development and that phytochemicals that are found naturally in fruits and vegetables may mediate a number of tumor targets [2], [3]. Clinical applications have been suggested for these dietary bioactive molecules because of their great ability to inhibit, retrieve, or delay multiple carcinogenic steps [4], [5], [6].

Resveratrol (3,5,4′-trihydroxy-*trans*-stilbene), which was first described in 1940, is a natural polyphenol that is found in a wide variety of plants, including grapes, berries, and peanuts. This molecule has been classified as a phytoalexin because it is synthesized by plants in response to a variety of environmental stress conditions such as fungal infections and UV radiation [7], [8]. Resveratrol is found in both *cis-* and *trans-*stereoisomeric forms; the *trans*-isomer is the form that is associated with most of the molecule's biological activities. Resveratrol as a dietary compound has been described as a powerful agent that is capable of preventing cardiovascular, inflammatory, and neurodegenerative diseases, including obesity and diabetes [7], [9]. The molecule is also able to modulate a wide spectrum of molecular targets, including those that are involved in cancer signaling pathways [8], [10].

The anticancer potential of resveratrol was recognized in 1997, when its ability to inhibit all three stages of carcinogenesis (i.e., initiation, promotion, and progression) was first demonstrated [10]. Subsequent studies have shown that the molecule exhibits anticancer activity when tested in various experimental models [11], [12]. Several studies have suggested that the mechanisms responsible for the chemopreventive properties of the molecule are closely associated with its antioxidant and anti-inflammatory properties [13].

Resveratrol is associated with the regulation of multiple cellular pathways, including the inhibition of carcinogen activation, the stimulation of carcinogen detoxifying enzymes, the modulation of proteins that are involved in cancer progression and apoptosis [14], [15], [16], and the inhibition of angiogenesis [17]. Studies also suggest that resveratrol can be used to sensitize tumors to specific cancer chemotherapeutics drugs [18]. Collectively, these findings provided sufficient evidence for the performance of clinical trials to investigate the chemopreventive and chemotherapeutic properties of this bioactive compound [4], [12].

p53 is a tumor-suppressor protein that plays an essential role in preventing cancer development by inducing cell cycle arrest or apoptosis in response to genotoxic stress. This protein is regulated primarily by the ubiquitin ligase MDM2, which binds to p53 and targets it for degradation in proteasomes. Normal p53 function is lost or inactive in approximately 50% of human cancers [19], [20], [21], [22].

Resveratrol is able to induce p53-dependent cell death in the JB6 mouse epidermal cell line [23], and the involvement of p53 in the anti-carcinogenic function of resveratrol has been established in a wide range of cells [7], [16], [23], [24], [25], [26]. Several studies have indicated that apoptosis occurs only in cells that express wild-type p53 but not in p53-mutated or p53-deficient cells [23], [27]. The precise role of resveratrol in p53-negative tumor cell lines, which may be resistant to conventional chemotherapeutic agents as a result of the loss of p53 expression or function, has not been extensively studied [28], [29], [30]. It is crucial to establish whether the transfection of wild-type p53 is able to restore the pro-apoptotic effects of resveratrol for the purpose of evaluating further gene therapy techniques aimed at fixing a faulty p53 molecule. Several studies of p53 gene therapy have demonstrated the efficiency of this approach not only in tumor cell lines but also in clinical trials [31].

In the present study, we investigated the effects of resveratrol in a cell line that carries a partially deleted p53 gene, and we tested the hypothesis that the transient transfection of p53 would cause these cells to become capable of responding to the pro-apoptotic properties of resveratrol. To directly investigate this issue, we transfected H1299 cells, which do not express the p53 gene, and compared the results with those obtained in MCF-7 human breast cancer, a cell line that has been extensively studied and that constitutively expresses the wild-type p53 gene.

We found that the transient transfection of wild-type p53-GFP gene causes H1299 cells to become more sensitive to resveratrol and responsive to its pro-apoptotic properties, similarly to findings in MCF-7 cells. Our results suggest that the application of resveratrol, in combination with other methods of promoting p53 activity in cells, such as gene therapy using wild-type p53 gene or chemicals that restore p53 function, is a promising new therapeutic strategy for cancer treatment.

## Materials and Methods

### 1. Reagents

All reagents used in this study were of analytical grade. Annexin V/Propidium Iodide Apoptosis Assay kit, bovine insulin, MTT (3,4,5-dimethiazol-2,5-diphenyltetrazolium bromide), phenylarsine oxide, pifithrin-α, PMSF (phenylmethylsulfonyl fluoride), PRIMA-1, protease inhibitors cocktail (#8340), and *trans-*resveratrol (3,5,4′-trihydroxy-*trans*-stilbene) were purchased from Sigma Aldrich (St. Louis, MO, USA). Okadaic acid and BAX inhibitor peptide (V5) were from Roche Applied Science (Indianapolis, IN, USA). Z-VAD-FMK [Z-Val-Ala-Asp(OMe)-CH2F] was from Calbiochem (San Diego, CA, USA). Z-LEHD-FMK was from R&D Systems (Minneapolis, MN, USA). DMEM (Dulbecco's Modified Eagle's Medium), RPMI-1640 medium, penicillin, and streptomycin were from Invitrogen (Carlsbad, CA, USA).

### 2. Cell culture

Human breast carcinoma cell line MCF-7 (p53 wild-type), human non-small lung carcinoma cell line H1299 (p53 negative), adenocarcinomic human alveolar basal epithelial cell line A549 (p53 wild-type), and human non-small lung carcinoma cell line H460 (p53 wild-type) were obtained from American Type Culture Collection (ATCC; Manassas, VA, USA). MCF-7 cells were cultured in DMEM containing 1.0 g/L glucose, supplemented with 2.0 g/L HEPES, 3.7 g/L sodium bicarbonate, 10% fetal bovine serum, and 5 µg/mL bovine insulin. H1299, A549 and H460 cells were cultured in RPMI-1640 containing 2.0 g/L glucose, supplemented with 2.0 g/L HEPES, 1.5 g/L sodium bicarbonate, and 10% fetal bovine serum. Peripheral blood mononuclear cells (PBMC) were isolated and cultured as described previously [32]. Penicillin (100 U/mL) and streptomycin (100 µg/mL) were added to culture plates prior to treatment with resveratrol. Cells were maintained at 37°C in a humidified atmosphere containing 5% CO_2_.

### 3. MTT reduction cell viability assay

Cells at 70–80% confluence were subcultured into a 24-well plate and treated with *trans-*resveratrol diluted in DMSO for 5 min, 24 h or 48 h. The culture medium was then removed, cells were washed with phosphate-buffered saline (PBS), and 0.5 mg/mL MTT was added. After 3 h at 37°C in a humidified atmosphere containing 5% CO_2_, MTT was carefully aspirated and the formazan produced was dissolved with 0.4 M acid-isopropanol. Controls were performed by adding 0.5% DMSO to plates. Cell viability was measured as the difference between the absorptions at wavelengths of 570 vs. 650 nm [33].

### 4. Western blotting analysis

To prepare whole cell extracts for western blotting, the culture medium was removed, and cells were washed twice with PBS and lysed with liquid nitrogen in a buffer containing 5 mM Tris-HCl pH 7.4, 10 mM EDTA, 1 mM Na_3_VO_4_, 5 mM NaF, 1 mM phenylarsine oxide, 1 µM okadaic acid, 1 mM PMSF, and a protease inhibitor cocktail. The protein concentration was determined as described by Lowry *et al*. (1951) [34], using bovine serum albumin as a standard. Cell lysates (100 µg) were resolved by SDS-PAGE (10%), and the separated proteins were transferred to polyvinylidene difluoride (PVDF) membranes. The membranes were blocked overnight at 4°C in Tris-buffered saline containing 1% Tween 20 (TBS-T) and 5% nonfat milk and incubated for 2 h with the primary antibody (1∶10000). The following antibodies were used: anti-p53 (DO-1), anti-MDM2 (D-7), and anti-GAPDH (0411) from Santa Cruz Biotechnology, Inc. (Santa Cruz, CA, USA) and anti-caspase 9 (#9502), anti-caspase 7 (#9492), anti-PARP (#9542), anti-phospho-Chk2 (Thr68) (#2661), and anti-p21 (#2946) from Cell Signaling Technology (San Diego, CA, USA). The membranes were washed with TBS-T and incubated with a peroxidase-conjugated secondary antibody (1∶5000) for 1 h. Immunoreactive bands were visualized by an ECL Western Blotting Detection System (GE Healthcare, Buckinghamshire, UK) according to the manufacturer's instructions. GAPDH (glyceraldehyde 3-phosphate dehydrogenase) levels in the cell lysates were used as a control. The densitometric quantification of bands was performed using ImageJ software, version 1.43r (NIH, USA).

### 5. Reverse transcriptase-polymerase chain reaction (RT-PCR)

For the semi-quantitative determination of p53 mRNA expression, total RNA was extracted and purified from H1299 and MCF-7 cells using the Illustra RNAspin Mini Kit (GE Healthcare, Buckinghamshire, UK), according to the manufacturer's instructions. The RNA concentrations were measured using a NanoDrop Spectrophotometer ND-1000 (Thermo Scientific, Waltham, MA, USA). Overall, 4 µg total RNA was used for cDNA production with the following primers: p53 fwd: 5′-GCT TCT TGC ATT CTG GGA CAG-3′; p53 rev: 5′-CTT CTT TGG CTG GGG AGA GG-3′ (626bp); GAPDH fwd: ATC ACC ATC TTC CAG GAG GCG; GAPDH rev: CCT GCT TCA CCA CCT TCT TG (574bp). The densitometric quantification of bands was determined using ImageJ software, version 1.43r.

### 6. H1299 cell transfection

The full-length p53-EGFP plasmid was obtained from Genscript Corp. (Piscataway, NJ, USA). The transient transfection experiments were performed using Fugene (Roche Diagnostics, Indianapolis, IN, USA) according to the manufacturer's instructions. For the analysis of GFP-p53, cells were plated onto glass-bottom dishes 48 h prior to the start of treatment. Cells were labeled with 10 µg/mL Hoechst 33342 (Molecular Probes, Eugene, OR, USA) for 30 min and washed three imes with PBS [35]. Images were collected using a confocal microscope (LSM Meta 510, Carl Zeiss, Oberkochen, Germany) and were analyzed using the Zeiss LSM Image Browser software program, version 4,2,0121.

### 7. Annexin V/propidium iodide apoptosis assay

Cells at 70–80% confluence were plated onto 24-well glass-bottom dishes and the assays were performed using the Annexin V/Propidium Iodide Apoptosis Assay kit according to the manufacturer's instructions. Images were collected using a confocal microscope (LSM Meta 510, Carl Zeiss, Oberkochen, Germany). To obtain the images the following wavelengths were used: annexin V-FITC (EX/EM ∼488 nm/∼540 nm) and propidium iodide (EX/EM ∼495 nm/∼635 nm). Images were analyzed using the Zeiss LSM Image Browser software program, version 4,2,0121.

### 8. TUNEL assay

Terminal deoxynucleotidyl transferase-mediated dUTP nick end labeling (TUNEL) was performed using a Click-iT® TUNEL Alexa Fluor® Imaging Assay kit (Invitrogen, Carlsbad, CA, USA) according to the manufacturer's instructions. Positive controls were obtained by the treatment of cells with deoxyribonuclease I, according to the manufacturer's instructions. The samples were analyzed using a confocal fluorescence microscope (Carl Zeiss International, Oberkochen, Germany). Images were analyzed using the Zeiss LSM Image Browser, version 4,2,0121.

### 9. Statistical analysis

The results were expressed as the mean ± standard error of the mean (S.E.M.). The data analysis and non-linear regressions were performed using the SigmaPlot software program (v. 10.0, Systat Inc., CA, USA) integrated with SigmaStat software (v. 3.2, Systat Inc. CA, USA). Student's *t*-test was used to compare means, and *p*<0.05 was considered to be statistically significant.

## Results

### 1. Resveratrol reduces human breast and lung cancer cell viability in a time- and dose-dependent manner

**Figure 1:**
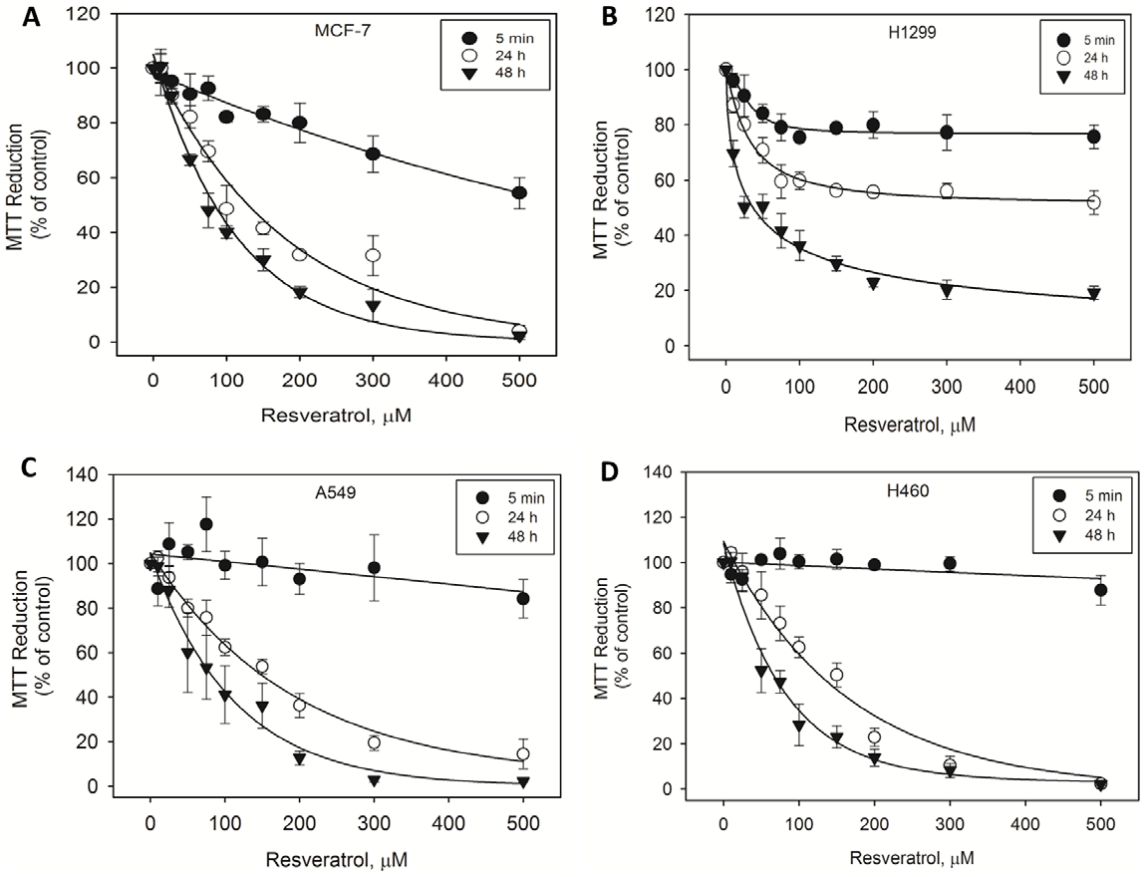
Time- and dose-dependent cytotoxicity induced by resveratrol in H1299 and MCF-7 cells. MCF-7 (A), H1299 (B), A549 (C), and H460 cells (D) were treated with various concentrations of *trans*-resveratrol (10–500 µM) diluted in DMSO for 5 min, 24 h and 48 h. The cell viability was measured by the MTT assay. The final concentration of DMSO in the culture medium was 0.5%. The results (n = 4) are expressed as a % of the control value, and the data presented are mean ± S.E.M.

Because p53 has been suggested to play a role in the anticancer properties of resveratrol, we tested the cytotoxic effects of this compound in the MCF-7 breast cancer, A549 lung cancer and H460 lung cancer cell lines (which express wild-type p53) and in the p53-deficient non-small lung cancer cell line H1299. As expected, p53 mRNA expression was confirmed by RT-PCR in the p53-positive cell lines but not in H1299 cells (data not shown). To test whether resveratrol had a cytotoxic effect, each of the cell lines was cultured with various resveratrol concentrations ranging from 10 to 500 µM for 5 min, 24 h, and 48 h. Cell viability was measured by the MTT reduction assay. Resveratrol reduced MCF-7, A549, H460 and H1299 cell viability in a time- and dose-dependent manner (Fig. 1). The sensitivity of MCF-7, A549 and H460 cells was greater than that of H1299, particularly when the cells were exposed for 24 h to resveratrol concentrations higher than 100 µM. Exposure to 500 µM resveratrol for 24 or 48 h reduced the viability of MCF-7 cells to nearly zero (Fig. 1A). Very similar profiles were observed for A549 and H460 cells (Fig. 1C and 1D). In contrast, approximately 50% and 20% of the H1299 cells remained viable at 24 and 48 h, respectively (Fig. 1B). The DMSO vehicle (0.5%) did not affect the viability of control cells (data not shown). We also tested the effects of resveratrol on peripheral blood mononuclear cells (PBMC), a less transformed cell line compared with the cancer cell lines studied. Resveratrol displayed a non-significant effect on PBMC viability (Fig. S1), suggesting that the cytotoxic effect of this compound is specific to cancer cell lines.

### 2. Resveratrol-induced cytotoxicity in MCF-7 cells is mediated by p53 activation

**Figure 2:**
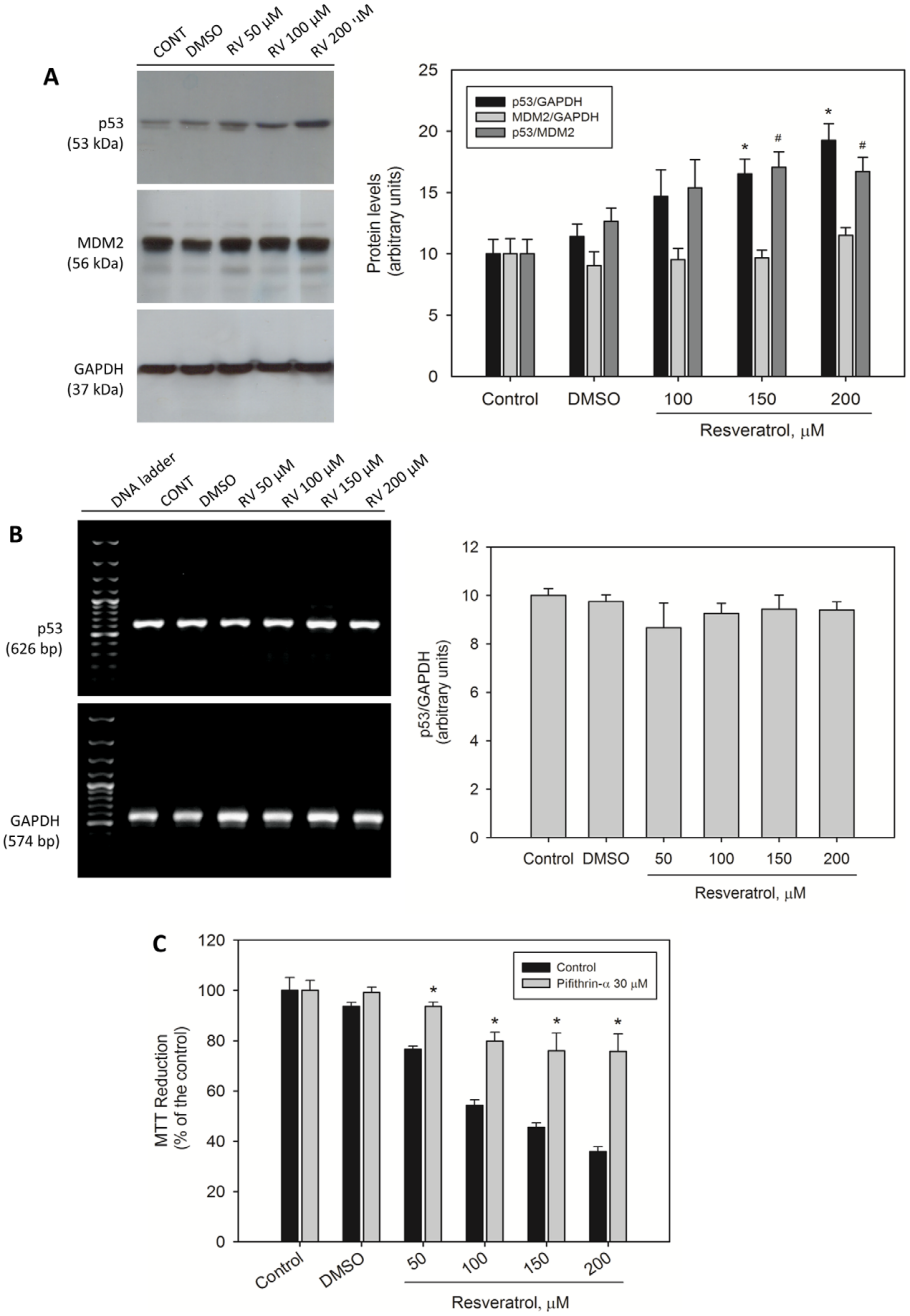
Resveratrol-induced cytotoxicity in MCF-7 cells is mediated by p53 activation. Effects of resveratrol on p53 and MDM2 protein levels (A) and on p53 mRNA levels (B) in MCF-7 cells. The cells were exposed to various concentrations of resveratrol or DMSO (0.5%) for 24 h. The protein levels were determined by western blotting analysis as described in Materials and Methods. GAPDH expression was used as a control. In panels A and B the data represent 3–5 independent experiments, and the band intensities are graphically represented with each group of images. (C) MCF-7 cell viability in the presence of the p53-specific inhibitor pifithrin-α. The cells were exposed to 30 µM pifithrin for 1 h prior to treatment with various *trans*-resveratrol concentrations (50–200 µM). The cell viability was measured by the MTT assay. The results (n = 3) are expressed as a % of the control, and the data shown are mean ± S.E.M. *p<0.05 in comparison with the respective control (Student's *t*-test).

The cellular levels of p53 were investigated in resveratrol-treated MCF-7 cells. The cells were exposed to various concentrations of resveratrol for 24 h, and the levels of p53 and MDM2 were determined by western blotting analysis (Fig. 2A). Resveratrol at concentrations of 100 and 200 µM produced a significant (*p*<0.05) dose-dependent increase in the total level of p53. This increase was not accompanied by an increase in the cellular level of MDM2. The p53/MDM2 ratio, as determined by densitometric quantification, revealed that the elevated levels of p53 following stimulation by resveratrol were not accompanied by an increase in MDM2-mediated protein degradation. p53 mRNA levels did not change when MCF-7 cells were treated with resveratrol under the same experimental conditions (Fig. 2B), suggesting that the increase in cellular levels of the p53 protein were not due to a stimulation of p53 gene expression.

**Figure 3:**
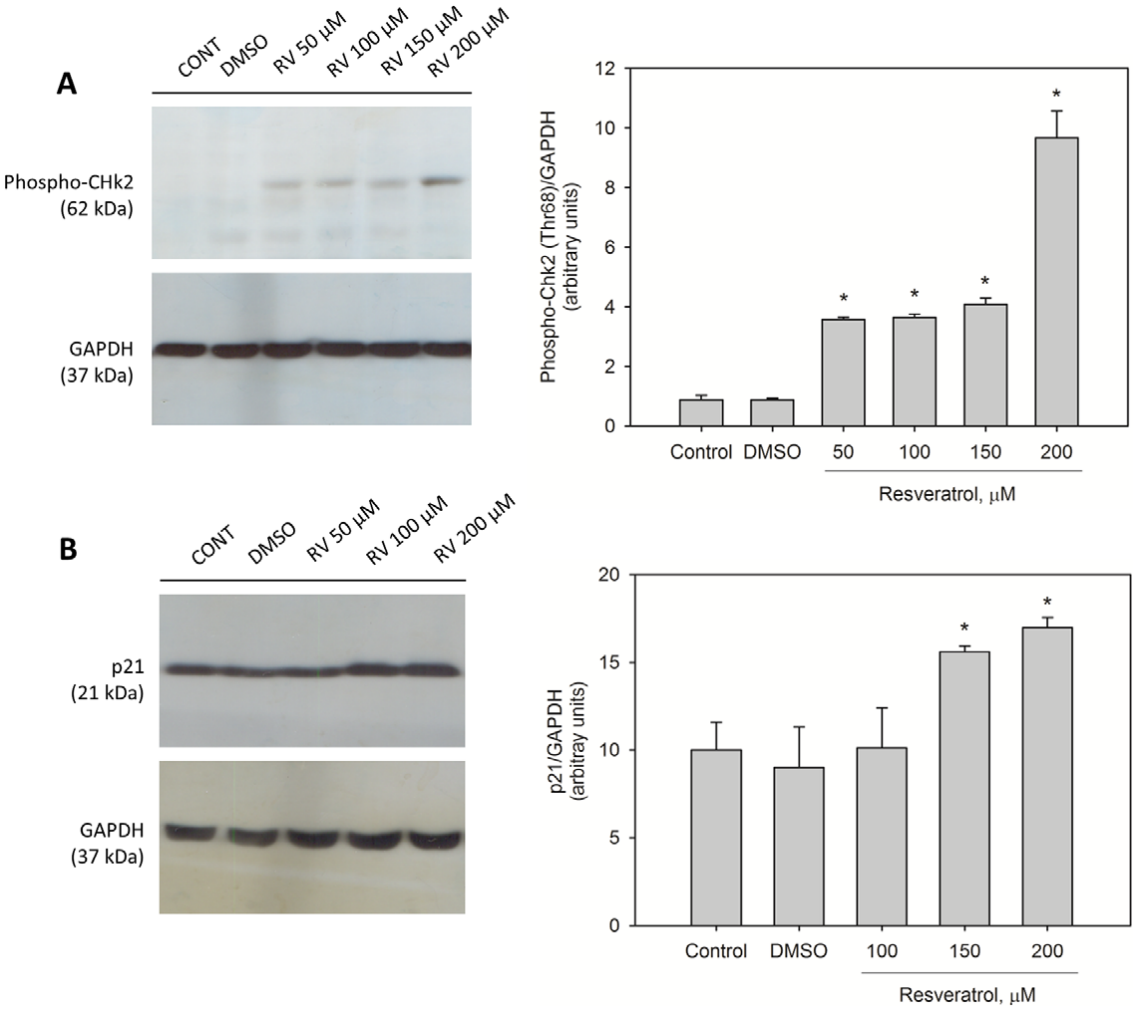
Activation of phospho-Chk2 and p21 by resveratrol in MCF-7 cells. Effects of resveratrol on phospho-Chk2 (A) and on p21 protein levels (B) in MCF-7 cells. The cells were exposed to various concentrations of *trans-*resveratrol or DMSO (0.5%) for 24 h. The protein levels were determined by western blotting analysis as described in Materials and Methods. GAPDH expression was used as a control. The data are representative of 3 independent experiments, and band intensities are graphically represented with each group of images. ^*^, *p*<0.05 in comparison with the respective control (Student's *t*-test).

A resveratrol-mediated increase of p53 levels was not observed when the exposure of cells to this compound for 24 h was followed by the removal of resveratrol from the culture medium for an additional 24 h period (data not shown). The exposure of MCF-7 cells to 200 µM resveratrol promoted the phosphorylation of checkpoint-2 protein (Chk2, a protein involved in several cellular processes including p53 activation in response to DNA damage) at Thr68 (Fig. 3A) and increased the cellular level of p21, which is one of the major p53-responsive genes (Fig. 3B).

To determine whether resveratrol-induced MCF-7 cell death is mediated by p53, the MTT reduction assay was performed in the presence of pifithrin-α, a specific p53 inhibitor that blocks the transcription of p53-responsive genes and also blocks p53-mediated apoptosis [36] (Fig. 2C). Cells were exposed to pifithrin-α 30 µM for 1 h and then treated with various concentrations of resveratrol for 24 h. We observed a greater number of viable cells under these conditions (Fig. 2C, gray bars) than in the absence of pifithrin-α (Fig. 2C, black bars), suggesting the possible involvement of p53 in resveratrol-induced cell death.

**Figure 4:**
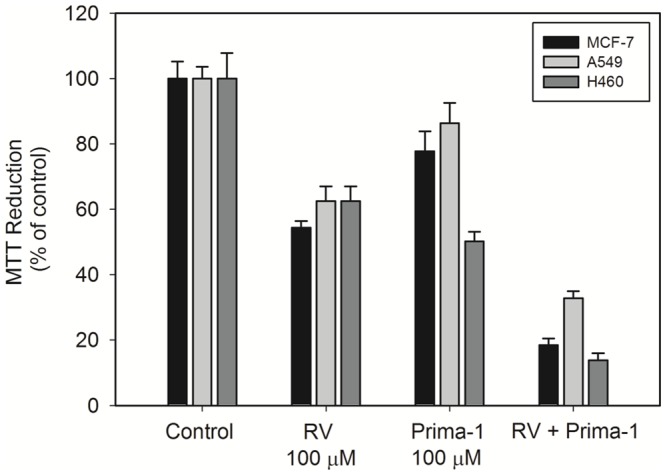
PRIMA-1 potentiates resveratrol-induced cytotoxicity in MCF-7, A549 and H460 cells. The cells were exposed for 24 h to 100 µM *trans-*resveratrol, 100 µM PRIMA-1, or 100 µM *trans-*resveratrol plus 100 µM PRIMA-1 100 µM. The data represent 3 independent experiments.

We also investigated the effects of PRIMA-1, a p53 activator drug, on the p53-positive cancer cell lines. PRIMA-1 at a concentration of 100 µM reduced cell viability, and the combination of this drug with 100 µM resveratrol potentiated the cytotoxic effect of resveratrol in MCF-7, A549 and H460 cells (Fig. 4).

### 3. Resveratrol does not induce apoptosis in H1299 p53-null cells

**Figure 5:**
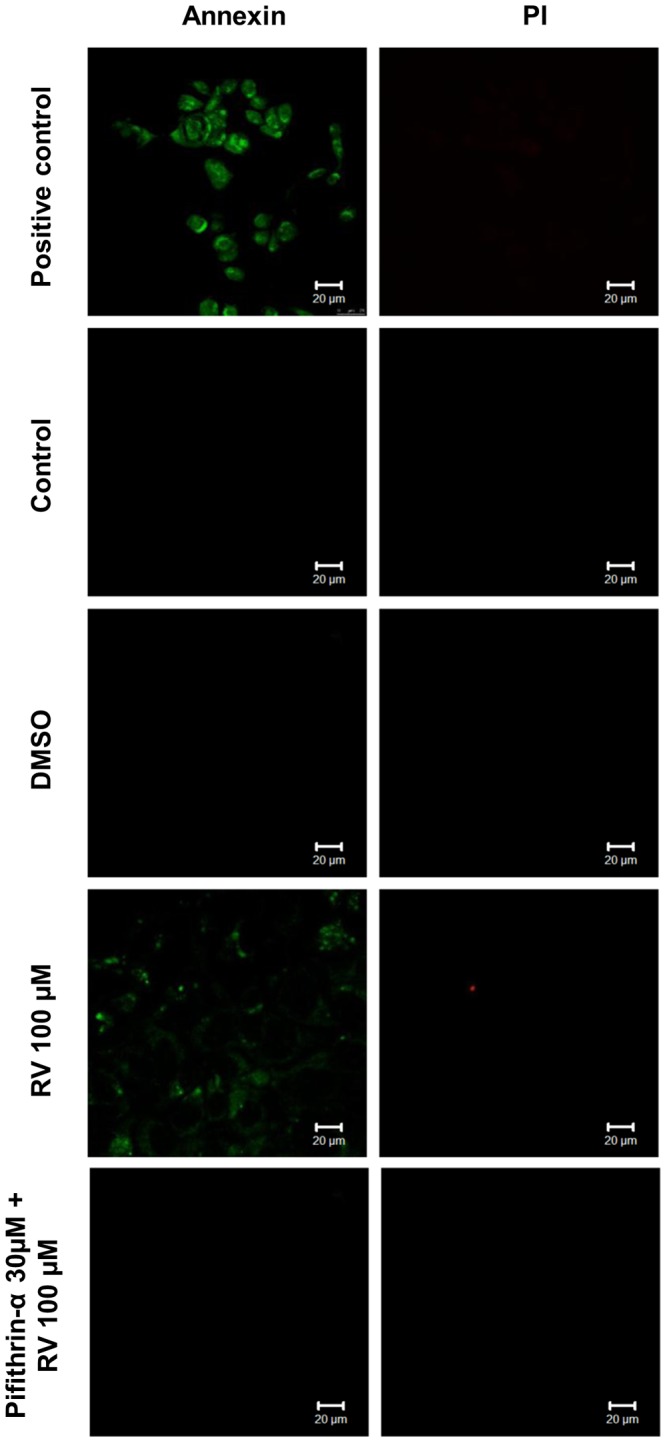
Resveratrol-induced apoptosis of MCF-7 cells is mediated by p53. The cells were treated with *trans-*resveratrol (100 µM) or DMSO (0.5%) and assayed using the Annexin V/Propidium Iodide Apoptosis Assay kit (Sigma Aldrich, St. Louis, MO, USA) according to the manufacturer's instructions. In one experiment, cells were exposed to 30 µM pifithrin for 1 h prior to treatment with *trans*-resveratrol. Images were collected by confocal microscopy (LSM Meta 510, Carl Zeiss, Oberkochen, Germany). Annexin V-FITC fluorescence: EX/EM ∼488 nm/∼540 nm; and Propidium Iodide fluorescence: EX/EM ∼495 nm/∼635 nm. Each image shown is represents two independent experiments.

**Figure 6:**
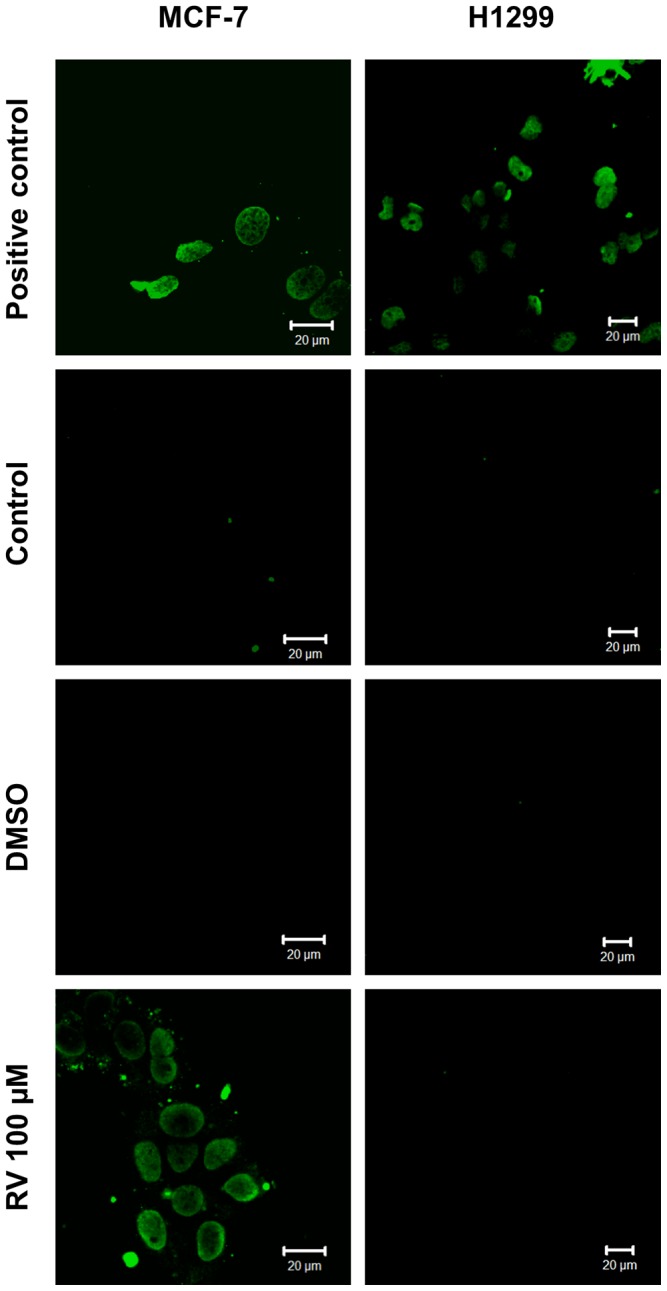
Resveratrol induces apoptosis in MCF-7 cells but not in H1299 cells. The cells were treated with *trans-*resveratrol (100 µM) and labeled using a Click-iT® TUNEL Alexa Fluor® Imaging Assay kit (Invitrogen, Carlsbad, CA, USA). TUNEL fluorescence: EX/EM ∼495 nm/519 nm. Each image represents two independent experiments.

**Figure 7:**
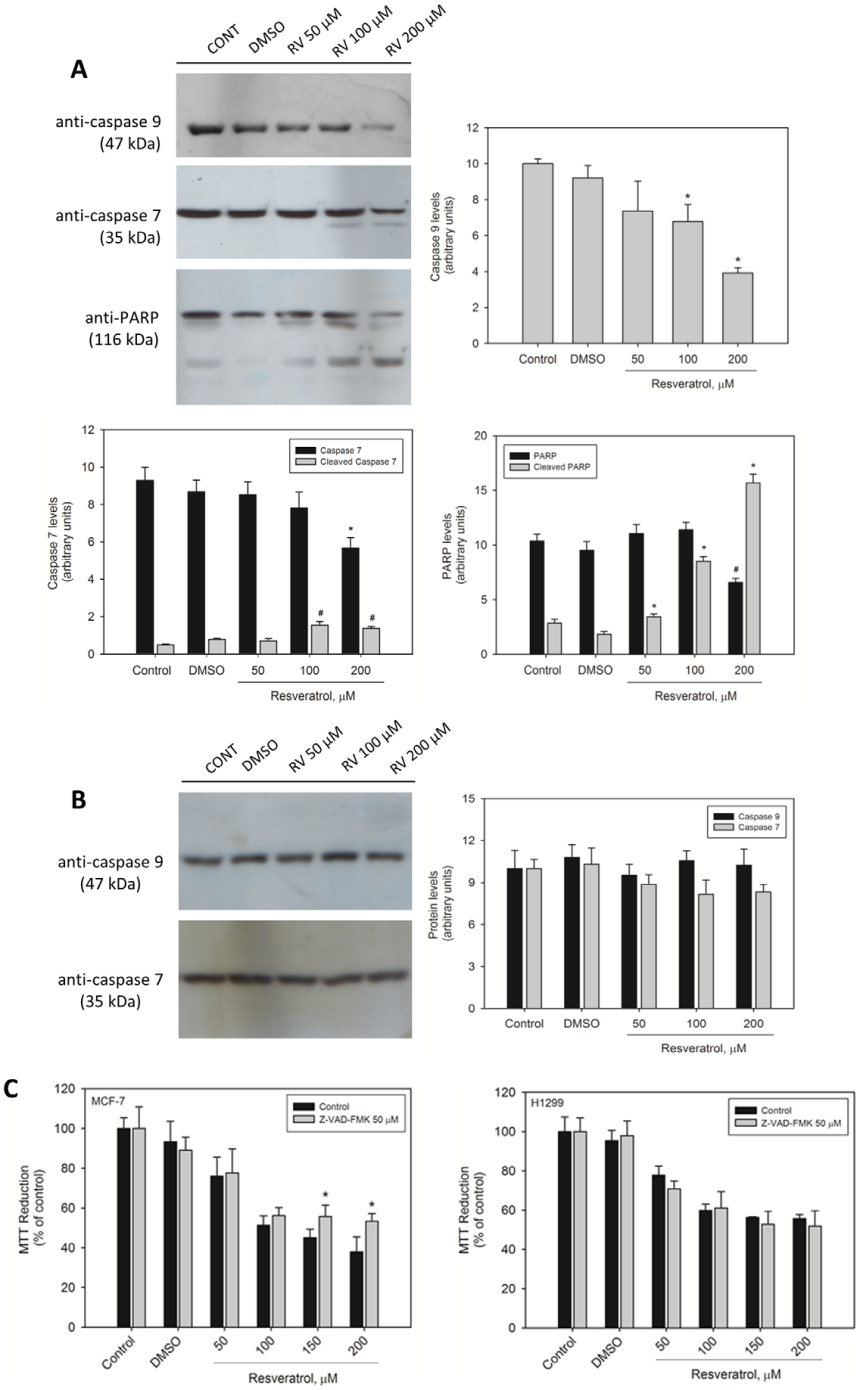
The effect of resveratrol on the proteins involved in MCF-7 and H1299 cell apoptosis. (A) Activation of caspase 9, caspase 7, and PARP by resveratrol in MCF-7 cells. (B) Levels of caspase 9 and caspase 7 in H1299 cells exposed to resveratrol. For western blotting analysis, the cells were exposed to various concentrations of resveratrol. The data represent 3–4 independent experiments, and the band intensities are graphically represented with each group of images. (C) MCF-7 and H1299 cells were pre-incubated with 50 µM of the caspase inhibitor Z-VAD-FMK for 1 h prior to treatment with various concentrations of *trans*-resveratrol. The cell viability was measured by the MTT assay. The results (n = 3) are expressed as % of control, and the data shown are mean ± S.E.M. *p<0.05 in comparison with the respective control (Student's *t*-test).

To test whether resveratrol-induced MCF-7 cell death is apoptosis-mediated, we performed an annexin V/propidium iodide assay. Resveratrol at 100 µM induced phosphatidylserine externalization (a process that indicates apoptosis), as shown by the annexin V-labeled cells in Figure 5. This effect was abrogated in the presence of pifithrin-α, indicating that p53-mediated apoptosis was occurring in the cells. Propidium iodide-labeled cells were not detected under the same conditions. A TUNEL assay was also performed in MCF-7 and H1299 cells. Resveratrol-induced apoptotic cell death was observed in MCF-7 cells but not in H1299 cells (Fig. 6). Resveratrol-induced apoptosis in MCF-7 cells was accompanied by a significant proteolytic cleavage of caspase 9 and caspase 7 (Fig. 7A). Poly (ADP) ribose polymerase was cleaved into two small fragments by exposure to increasing concentrations of resveratrol. No activation of caspases 9 and 7 was detected in p53-negative H1299 cells (Fig. 7B). To test the possibility that resveratrol-induced cell death is mediated by caspase signaling pathways in MCF-7 but not in H1299, we performed an MTT reduction assay in the presence of the pan caspase inhibitor Z-VAD-FMK (Fig. 4C). Cells were exposed to 50 µM Z-VAD-FMK for 1 h and then treated with various concentrations of resveratrol for 24 h. The number of viable MCF-7 cells was significantly higher in the presence of 100 or 200 µM resveratrol (Fig. 7C, gray bars) than in the absence of inhibitor (Fig. 7C, black bars), suggesting that caspases are involved in resveratrol-induced cell death. Z-VAD-FMK did not affect the viability of H1299 cells under the same conditions. The possible involvement of caspases was also investigated using the caspase 9-specific inhibitor Z-LEDH-FMK. MCF-7 but not H1299 showed a higher percentage of viable cells in comparison with control in the presence of Z-LEDH-FMK (Fig. S2).

We examined the possible involvement of Bax in the resveratrol-induced apoptosis of MCF-7 cells by measuring the cellular levels of this protein and by applying a specific Bax peptide inhibitor (V5) following the treatment of cells with resveratrol. We observed higher Bax levels in cells treated with 100 µM resveratrol and higher numbers of viable cells in the presence of V5, suggesting the involvement of this pro-apoptotic protein in the MCF-7 cell death cascade (Fig. S3).

### 4. H1299 cells require p53 to undergo apoptosis in response to resveratrol exposure

**Figure 8:**
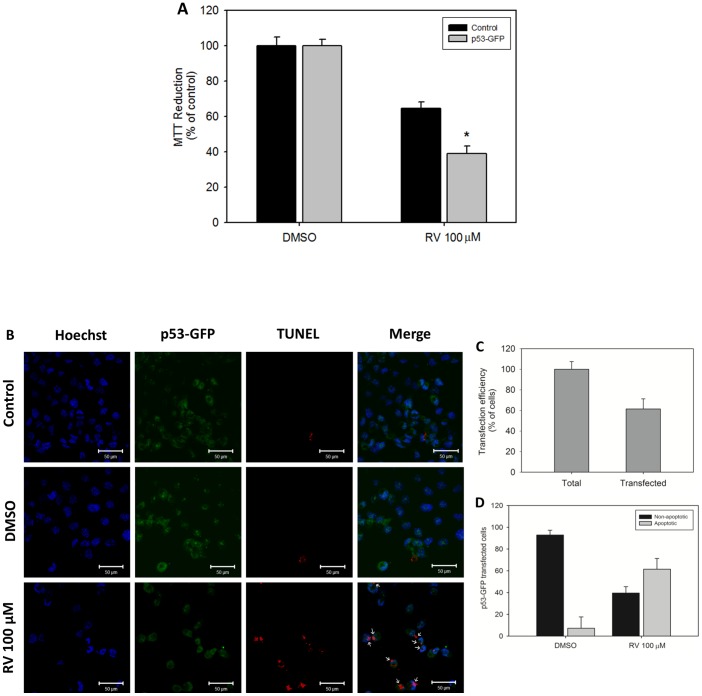
Resveratrol reduces the viability of p53-transfected H1299 cells by the induction of apoptosis. (A) p53-transfected and non-transfected H1299 cells were treated with resveratrol (100 µM). The cell viability was measured by the MTT assay. The results (n = 4) are expressed as % of control, and the data shown are mean ± S.E.M. *p<0.05 in comparison with the respective control (Student's *t*-test). (B) The cells were transiently transfected with the full-length p53-EGFP plasmid and treated with resveratrol (100 µM). The cells were then double-labeled with Hoechst 33342 (Molecular Probes, Eugene, OR, USA) and with Click-iT® TUNEL Alexa Fluor® Imaging Assay (Invitrogen, Carlsbad, CA, USA). Hoeschst fluorescence: EX/EM ∼350 nm/461 nm. TUNEL fluorescence: EX/EM ∼495 nm/519 nm. In the merged images, the arrows indicate cell death of p53-transfected cells. (C) The rate of transfection efficiency for H1299 cells. (D) The quantification of apoptotic and non-apoptotic transfected H1299 cells following exposure to resveratrol.

To determine whether the expression of p53 in H1299 cells causes the cells to become susceptible to the effects of resveratrol, we conducted transient transfection assays of wild-type p53-GFP plasmid. An MTT assay was performed in p53-transfected H1299 to test the cytotoxicity of resveratrol in these cells. Twenty-four hours after the transfection, the cells were treated with 100 µM resveratrol for an additional 24 h. The cytotoxic effect of resveratrol on H1299 cells was enhanced (i.e., the cells became more susceptible to this compound) through p53 expression (Fig. 8A). The effects of resveratrol on H1299 cells as well as on MCF-7 cells appear to be dependent on wild-type p53 function. The transient transfection of p53-GFP triggered resveratrol-induced apoptosis in H1299 (Fig. 8B). The rate of transfection efficiency obtained in these experiments was approximately 60% (Fig. 8C). Merged images from fluorescence microscopy (Fig. 8D) indicate that most of the p53-transfected cells were undergoing apoptosis (white arrows).

## Discussion

In this study, we examined the effects of resveratrol on the p53-deficient non-small human lung cancer cell line H1299 in comparison with the effects of resveratrol on cancer cell lines (human breast cancer MCF-7, lung cancer cell A549 and H460) that express wild-type p53. The hypothesis tested was that p53 transfection in H1299 cells, which carry a partially deleted p53 gene, makes these cells capable of responding to resveratrol pro-apoptotic properties. MCF-7, A549 and H460 cells were found to be more sensitive to resveratrol-induced cytotoxic effects than were H1299 cells. Resveratrol induced caspase-mediated apoptosis in MCF-7 cells but not in H1299 cells, and transient transfection of wild-type p53 rendered H1299 susceptible to the pro-apoptotic effects of resveratrol.

H1299 cells were more resistant to resveratrol, particularly at high concentrations, than were the p53-positive cancer cell lines. The lack of p53 expression in H1299 may account for its higher resistance to cell death in comparison with p53-positive MCF-7 cells. H1299 did not undergo apoptosis following exposure to resveratrol, in contrast to MCF-7. Other mechanisms of cell death may be related to this effect; *e.g*., a recent study indicated that resveratrol triggers an interaction of processes that occur during apoptosis, cell cycle arrest, and autophagy in tumor cells [37].

Resveratrol-induced cytotoxicity and apoptosis were mediated in part by p53 in MCF-7 cells, as indicated by the cell viability experiments performed in the presence of the p53-specific inhibitor pifithrin-α. The endogenous levels of p53 protein were significantly higher in the presence of resveratrol, which is in agreement with other reports [38], [39]. In the present study, the increase of p53 levels was not followed by an increase in MDM2 levels, suggesting that the high p53/MDM2 ratio reflected a lower ubiquitin-dependent p53 degradation level, most likely as a result of stabilizing mechanisms operating on the protein. The increase in p53 levels resulting from resveratrol exposure may be correlated with posttranslational protein modifications (phosphorylation or acetylation) because p53 gene expression, as revealed by RT-PCR, did not change. Resveratrol stimulated the phosphorylation of Chk2 protein at Thr68. This protein is involved in several cellular processes including p53 activation in response to DNA damage. Chk2 protein in this phosphorylated state is active and consequently able to stimulate p53 by phosphorylation. In contrast to the results of other studies [24], [40], we found that resveratrol did not change the phosphorylation of p53 at Ser15 (data not shown). However, we cannot rule out the possibility that Chk2 activates the p53 protein by phosphorylating a different amino acid residue or by other mechanisms. We also found that in the presence of PRIMA-1, resveratrol-induced cytotoxicity was potentiated in MCF-7, A549 and H460 cells. Although PRIMA-1 was originally known as a p53 reactivating drug that can rescue the function of mutant forms of p53 by interacting with p53 core domain [41], we found that this drug was able to increase cell death in all three tumor cell lines expressing wild-type p53. It is possible to speculate that PRIMA-1 may stabilize or activate wild-type p53 in this experimental context, considering that the protein can be expressed in a mutant-like conformation, which allows the interaction with PRIMA-1.

Resveratrol-induced apoptosis in MCF-7 was mediated by the proteolytic cleavage of the initiator caspase 9 and the executioner caspase 7. A recent study demonstrated that caspase 9 silencing inhibits resveratrol-induced caspase activation in LNCaP cells, which express wild-type p53 [42]. We found that poly (ADP) ribose polymerase, a protein that is involved in a variety of cellular processes including DNA repair, was cleaved into two small fragments by exposure to increasing concentrations of resveratrol. Caspase 3 is considered to be essential for PARP cleavage in cancer cells. MCF-7 strains generally do not express this protein [43]. Other caspases may therefore replace caspase 3 in regard to this function. Based on our observation that caspase 7 is activated in MCF-7 cells following exposure to resveratrol, we propose that PARP cleavage is mediated primarily by caspase-7. In contrast to MCF-7 cells, no active caspases were detected in p53-negative H1299 cells. These results are consistent with our finding that MCF-7 cells but not H1299 cells were positively labeled by TUNEL following exposure to 100 µM resveratrol. We found that the reduction in cell death that was promoted by the inhibitors of caspases in MCF-7 cells was lower than the reduction in cell death that was induced by the p53 inhibitor. Thus, we can speculate that in this context, p53 should not only be involved in apoptosis but also play a role in other mechanisms of cell death.

The effects of resveratrol on p53-negative cells are important in regard to the potential application of this compound to the various types of cancer cells that display deregulated tumor suppression pathways that are under p53 control. This type of deregulation, which is caused primarily by mutations and/or the formation of p53 amyloid-like aggregates, is one of the most common defects observed in human malignancies [44], [45], [46]. Although there have been several reports that the anticancer mechanisms triggered by resveratrol may be independent of p53 cellular status [27], [47], [48], we found that H1299 requires p53 expression to undergo apoptosis in response to resveratrol exposure.

Resveratrol is a bioactive compound that induces a multitude of effects in normal and cancer cell signaling pathways. These effects presumably depend on various cellular conditions and on the concentration and the duration of exposure to the compound. The resveratrol concentrations that were used in this study were higher than the concentrations that are typically found in the plasma of patients. Any extrapolation from results that are obtained under in vitro conditions should be viewed with caution because the active concentrations that are determined in vitro may vary several-fold under various culture conditions. Thus, these types of extrapolations are not straightforward because the in vivo growth and survival of cancer cells are influenced by complex interactions with populations of various types of normal cells (e.g., lymphocytes, macrophages, endothelial cells and stromal cells) that are not present in vitro. Furthermore, it is impossible to precisely reproduce the kinetic changes in the concentrations and exposure times of resveratrol and its metabolites that occur in vivo. We tested low doses of resveratrol (less than 5 µM) in MCF-7 cells for up to 72 h and did not observe any significant changes in cell viability (data not shown).

Several reports have shown that resveratrol does not damage non-malignant cells although it may trigger direct cytotoxic effects on cancer cells [49], [50], [51], [52]. Other reports indicate that resveratrol is cytotoxic to normal cells in certain cases [53]. We found that resveratrol at 50 to 200 µM had no significant cytotoxic effect on non-malignant PBMC (Fig. S1) or on BHK-21 cells (data not shown) after 24 hours of exposition. However, more studies focused on chronic resveratrol toxicity should be conducted.

The introduction of wild-type p53 into cancer cells that carry null or mutant p53 genes may suppress tumor formation by inducing apoptotic cell death [54]. We found that p53-transfected H1299 cells were susceptible to the pro-apoptotic properties of resveratrol, similarly to p53-positive MCF-7 cells. Certain clinical trials based on p53 gene therapy of cancer patients indicate that this is a promising therapeutic approach [31].

In summary, resveratrol is a potent phytochemical compound that, in combination with gene therapy using the wild-type p53 gene, may provide a promising new therapeutic strategy for cancer treatment.

## Supporting Information

Figure S1 **Resveratrol does not induce cytotoxicity in peripheral blood mononuclear (PBMC) cells.** Cells were treated with different *trans*-resveratrol concentrations diluted in DMSO for 24 h. Cell viability was then measured by MTT assay. The final concentration of DMSO in culture medium was 0.5%. Results (n = 3) are expressed as a % of the control and data are means ± S.E.M.
(TIF)

Figure S2 **Resveratrol-induced apoptosis is mediated by caspase 9 in MCF-7, but not in H1299 cells.** MCF-7 and H1299 cells were pre-incubated with 20 µM of the caspase 9 inhibitor Z-DH-FMK during one hour, prior to treatment with different *trans*-resveratrol concentrations. Cell viability was then measured by MTT assay. Results (n = 3) are expressed as % of control and data are means ± S.E.M. *p<0.05 when compared to controls (Student's *t*-test).
(TIF)

Figure S3 **Involvement of Bax in resveratrol-induced apoptosis.** (A) Cells were exposed to 100 µM of resveratrol or DMSO (0.5%) for 24 h. Protein levels were determined by western blotting analysis, as described in Materials and Methods. (B) Cells were pre-incubated with 50 µM of Bax inhibitor peptide (V5) during one hour, prior to treatment with different *trans*-resveratrol concentrations. Cell viability was then measured by MTT assay. Results (n = 3) are expressed as % of control and data are means ± S.E.M. *p<0.05 when compared to controls (Student's *t*-test).
(TIF)
