# Supplementary figures and images for: Transient Transfection of a Wild-Type p53 Gene Triggers Resveratrol-Induced Apoptosis in Cancer Cells
Source: PLoS One. 2012 Nov 12;7(11):e48746. doi: 10.1371/journal.pone.0048746 (PMC3495968; doi:10.1371/journal.pone.0048746)

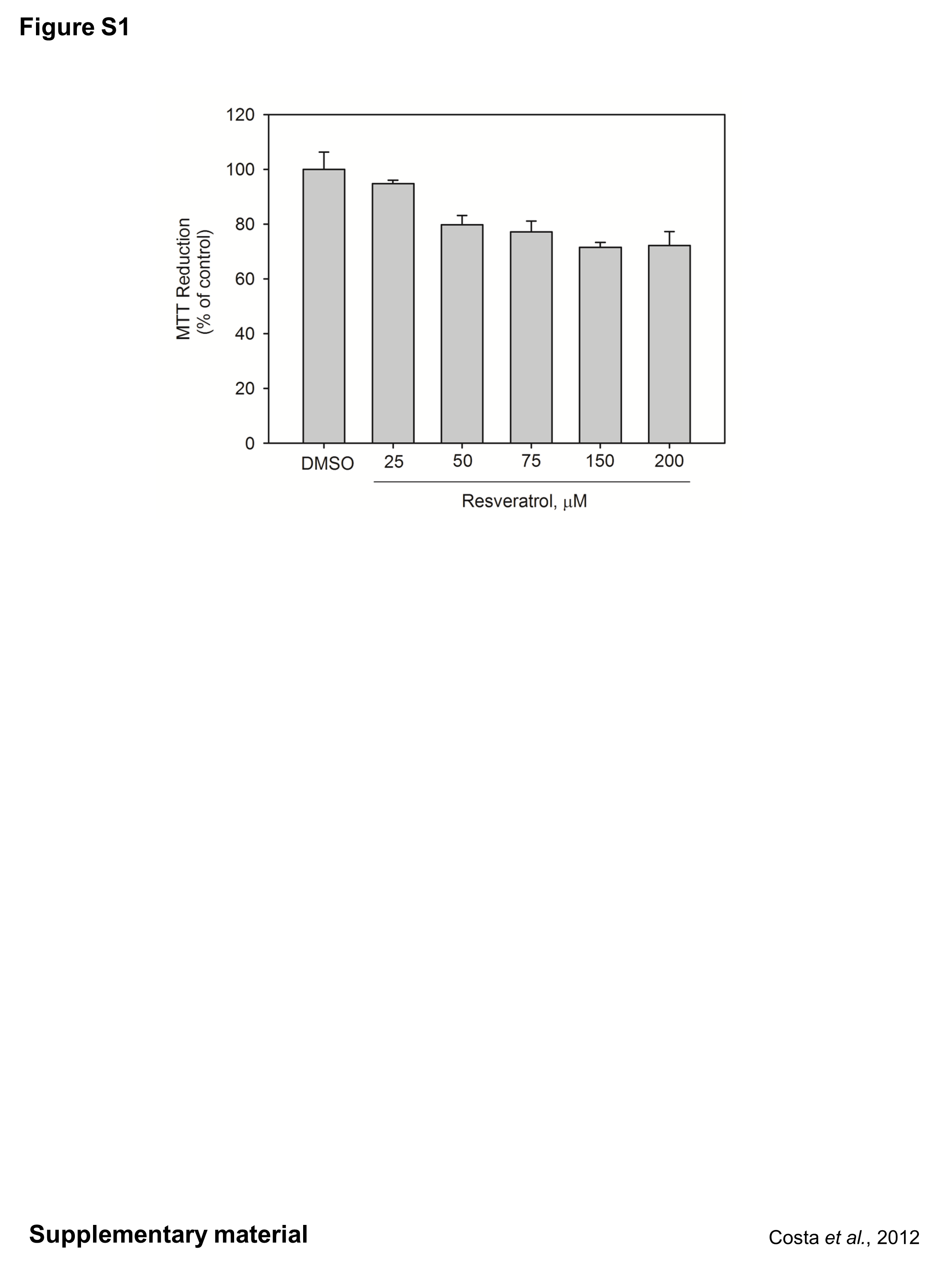

Supplement: Figure S1 — Resveratrol does not induce cytotoxicity in peripheral blood mononuclear (PBMC) cells. Cells were treated with different trans-resveratrol concentrations diluted in DMSO for 24 h. Cell viability was then measured by MTT assay. The final concentration of DMSO in culture medium was 0.5%. Results (n = 3) are expressed as a % of the control and data are means ± S.E.M. (TIF) [file pone.0048746.s001.tif]

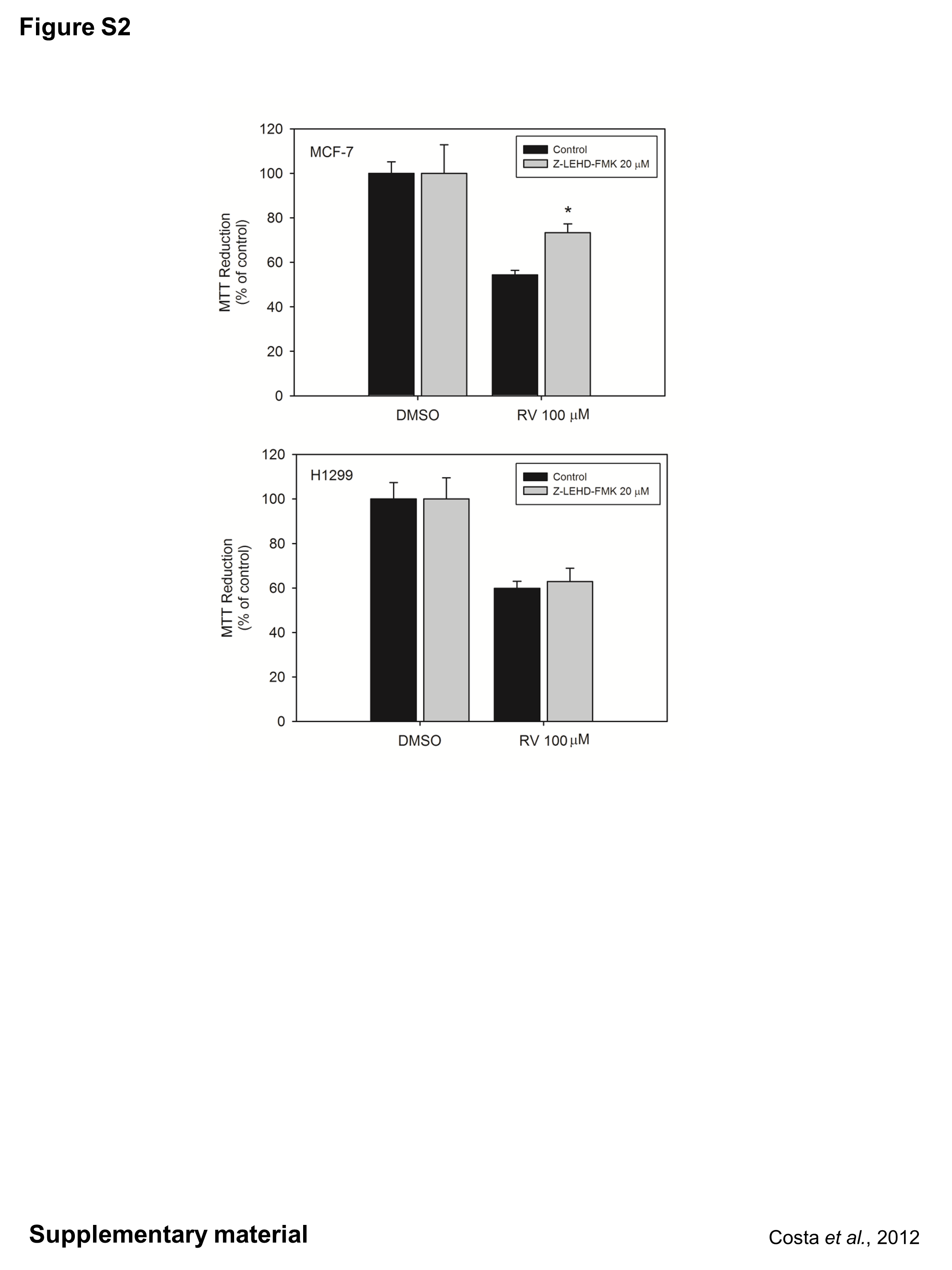

Supplement: Figure S2 — Resveratrol-induced apoptosis is mediated by caspase 9 in MCF-7, but not in H1299 cells. MCF-7 and H1299 cells were pre-incubated with 20 µM of the caspase 9 inhibitor Z-DH-FMK during one hour, prior to treatment with different trans-resveratrol concentrations. Cell viability was then measured by MTT assay. Results (n = 3) are expressed as % of control and data are means ± S.E.M. *p<0.05 when compared to controls (Student's t-test). (TIF) [file pone.0048746.s002.tif]

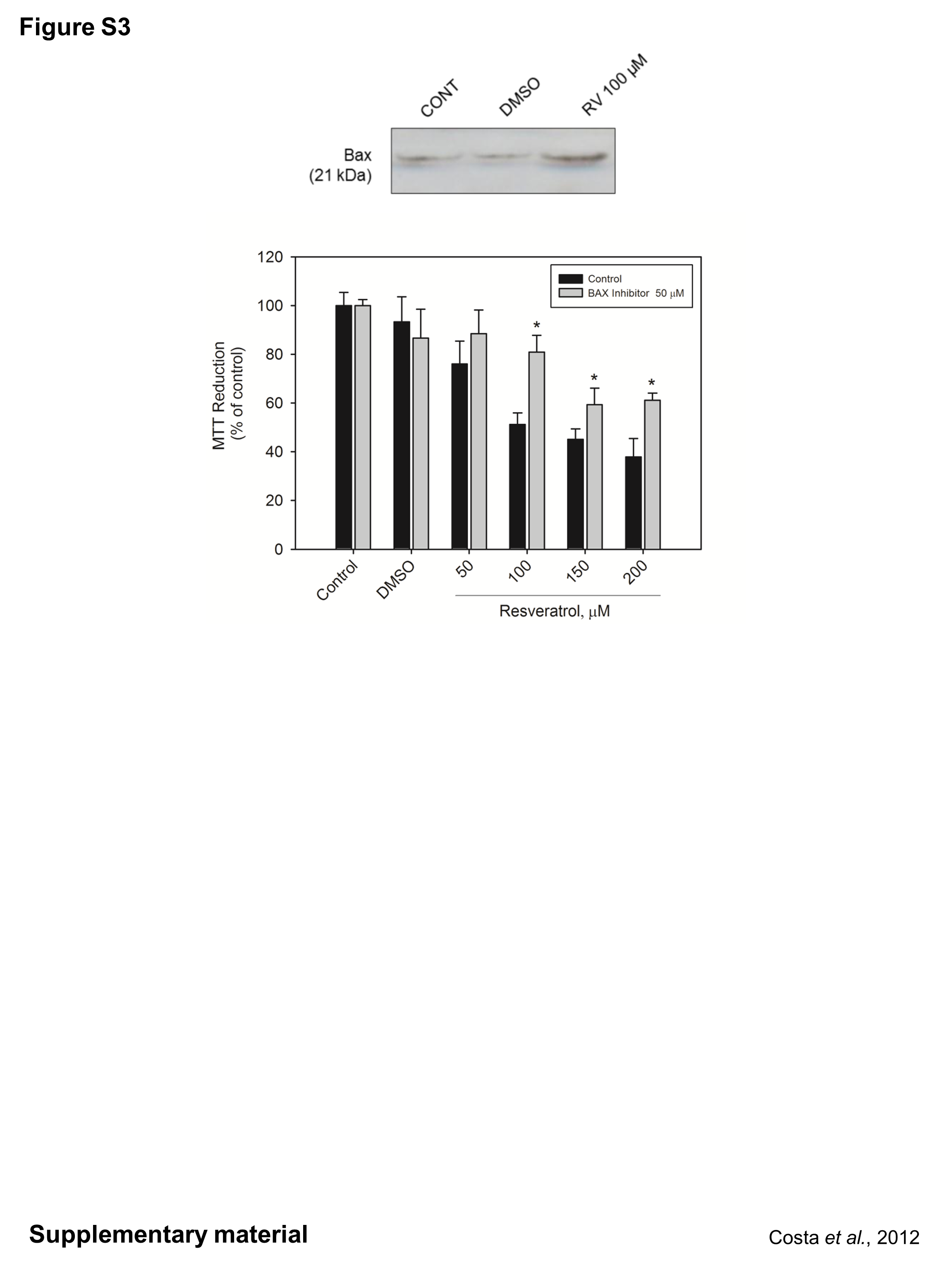

Supplement: Figure S3 — Involvement of Bax in resveratrol-induced apoptosis. (A) Cells were exposed to 100 µM of resveratrol or DMSO (0.5%) for 24 h. Protein levels were determined by western blotting analysis, as described in Materials and Methods. (B) Cells were pre-incubated with 50 µM of Bax inhibitor peptide (V5) during one hour, prior to treatment with different trans-resveratrol concentrations. Cell viability was then measured by MTT assay. Results (n = 3) are expressed as % of control and data are means ± S.E.M. *p<0.05 when compared to controls (Student's t-test). (TIF) [file pone.0048746.s003.tif]
